# Altered VEGF Splicing Isoform Balance in Tumor Endothelium Involves Activation of Splicing Factors Srpk1 and Srsf1 by the Wilms’ Tumor Suppressor Wt1

**DOI:** 10.3390/cells8010041

**Published:** 2019-01-11

**Authors:** Kay-Dietrich Wagner, Mounir El Maï, Michael Ladomery, Tareg Belali, Nathalie Leccia, Jean-François Michiels, Nicole Wagner

**Affiliations:** 1 Université Côte d’Azur, Institute of Biology Valrose, Nice (iBV), CNRS UMR7277, INSERM U1091, 06107 Nice, France; kwagner@unice.fr; 2 Université Côte d’Azur, Institute for Research on Cancer and Aging, Nice (IRCAN), CNRS UMR7284/INSERM U1081, 06107 Nice, France; elmai_mounir@yahoo.fr; 3 Faculty of Health and Applied Sciences, University of the West of England, Bristol BS16 1QY, UK; Michael.Ladomery@uwe.ac.uk (M.L.); Tareg.Belali@uwe.ac.uk (T.B.); 4 Department of Pathology, CHU Nice, 06107 Nice, France; leccia.n@chu-nice.fr (N.L.); michiels.jf@chu-nice.fr (J.-F.M.)

**Keywords:** VEGF isoforms, splicing factors, endothelium, transcriptional regulation, Wilms’ tumor suppressor Wt1

## Abstract

Angiogenesis is one hallmark of cancer. Vascular endothelial growth factor (VEGF) is a known inducer of angiogenesis. Many patients benefit from antiangiogenic therapies, which however have limitations. Although VEGF is overexpressed in most tumors, different VEGF isoforms with distinct angiogenic properties are produced through alternative splicing. In podocytes, the Wilms’ tumor suppressor 1 (WT1) suppresses the Serine/arginine-rich protein-specific splicing factor kinase (SRPK1), and indirectly Serine/arginine-rich splicing factor 1 (Srsf1) activity, and alters VEGF splicing. We analyzed VEGF isoforms, Wt1, Srpk1, and Srsf1 in normal and tumor endothelium. Wt1, Srpk1, Srsf1, and the angiogenic VEGF164a isoform were highly expressed in tumor endothelium compared to normal lung endothelium. Nuclear expression of Srsf1 was detectable in the endothelium of various tumor types, but not in healthy tissues. Inducible conditional vessel-specific knockout of Wt1 reduced Wt1, Srpk1, and Srsf1 expression in endothelial cells and induced a shift towards the antiangiogenic VEGF120 isoform. Wt1(−KTS) directly binds and activates both the promoters of Srpk1 and Srsf1 in endothelial cells. In conclusion, Wt1 activates Srpk1 and Srsf1 and induces expression of angiogenic VEGF isoforms in tumor endothelium.

## 1. Introduction

Tumor growth as well as development and organ homeostasis require vascular proliferation (reviewed in [1,2]). Vascular endothelial growth factor A (VEGF), which was initially identified as vascular permeability factor [3,4], is the best-known factor inducing endothelial cell proliferation and angiogenesis [5,6]. Normal VEGF levels are required for embryonic development [7,8,9,10,11] and anti-angiogenic therapies targeting VEGF are widely used for the treatment of cancers (reviewed in [12]).

VEGF transcription during tumor growth is stimulated by hypoxia [13,14], which is mediated via hypoxia inducible factor 1 (Hif-1) [14] and the Wilms’ tumor suppressor 1 (Wt1) [15,16]. Additional non-coding transcripts in the promoter region modulate expression of VEGF mRNA [17].

Besides the transcriptional regulation of VEGF by WT1, WT1 is also involved in Vegf RNA splicing in murine hematopoiesis [18] and in podocyte cell lines [19]. Different VEGF isoforms are generated via alternative splicing of exons 6, 7, and 8 of the VEGF gene resulting mainly in human VEGF 189, 165, and 121 variants (Vegf 188, Vegf 164, and Vegf 120 in mice, respectively). Pro-angiogenic isoforms (VEGF-A_xxx_) are generated by proximal and anti-angiogenic (VEGF-A_xxx_b) forms by distal splice site selection in exon 8 (reviewed in [1,20]). Lack of Wt1 in murine hematopoietic progenitor cells results in a shift towards the Vegf 120 isoform, which is associated with apoptotic cell death [18]. In human podocyte cell lines, WT1 binds and suppresses the promoter of the splicing factor kinase SRPK1. In WT1 mutant podocytes, SRPK1-mediated hyperphosphorylation of the RNA binding protein SRSF1 results in a shift from the anti-angiogenic VEGF 165b towards the pro-angiogenic VEGF 165a isoform [19].

Endogenous VEGF in endothelial cells is important to regulate key vascular proteins and maintain endothelial cell homeostasis [21], but little is known about VEGF isoform expression in endothelial cells from different organs, tumors, and their regulation. Therefore, we determined VEGF isoforms, Wt1, Srpk1, and Srsf1 expression in normal and tumor endothelial cells. We generated an inducible conditional endothelial cell-specific knockout mouse model for Wt1 to analyze the effect on Srpk1 and Srsf1 expression and Vegf isoform distribution.

We show that, in tumor endothelium, Wt1, Srpk1, and Srsf1 are upregulated compared to normal lung endothelium. Wt1 functions as direct activator of both Srpk1 and Srsf1, and affects Vegf isoform distribution in endothelial cells. Disrupting Wt1 in endothelial cells reduces Srpk1 and Srsf1 expression and alters Vegf isoform distribution, which might contribute to the antitumor activity upon targeting Wt1.

## 2. Materials and Methods

### 2.1. Animals

All animal work was conducted according to national and international guidelines and was approved by the local ethics committee (PEA-NCE/2013/106). *Wt1^Lox/Lox^* and *Tie2-CreERT2* animals were crossed to generate *Tie2-CreERT2*; *Wt1^Lox/Lox^* mice [22]. All animals were backcrossed four times onto the C57/BL6 genetic background. The genotype of animals was identified by PCR using the following oligonucleotides and PCR conditions: Cre-F 5′-CGCAGAACCTGAAGATGTTCGCGA-3′; Cre-B 5′-GGATCATCAGCTACACCAGAGACG-3′ (95 °C 3 min, [94 °C 20 s, 60 °C 45 s, 72 °C 1 min] × 27, 72 °C 7 min), Wt1lox-F 5′-TGGGTTCCAACCGTACCAAAGA-3′; Wt1lox-B 5′-GGGCTTATCTCCTCCCATGT-3′ (95 °C 3 min, [93 °C 45 s, 56 °C 45 s, 72 °C 45 s] × 35, 72 °C 7 min).

Age-matched *Tie2-CreERT2*; *Wt1^Lox/Lox^* male and female mice were injected for one week intraperitoneally with either sunflower oil (vehicle) or Tamoxifen dissolved in sunflower oil in a dose of 33 mg/kg per day [23]. Age-matched single *Tie2-CreERT2* transgenic animals injected with Tamoxifen served as additional controls for Cre and Tamoxifen effects. One week after the last Tamoxifen or vehicle treatment, 1 × 10^6^ B16F10 or LLC1 tumor cells were injected subcutaneously. Tumors and organs were collected after three to four weeks. C57/BL6 animals were used for isolation of endothelial cells from lungs or tumors. In these animals, tumors were induced by subcutaneous injection of 1 × 10^6^ LLC1 tumor cells.

### 2.2. Cell Culture

LLC1 mouse lung cancer cells (accession number CRL-1642) were grown in DMEM-F12 medium (Lonza, Levallois-Perret, France), C166 mouse endothelial cells (accession number CRL-2581), and B16-F10 mouse melanoma cells (accession number CRL-6475) in DMEM medium. Media were supplemented with 10% fetal calf serum (FCS), 100 IU/mL penicillin and 100 µg/mL streptomycin.

### 2.3. Endothelial Cell Isolation

Mouse lung and tumor endothelial cells (EC) were isolated from C57/BL6 mice as previously described [24,25]. Alternatively, B16 or LLC1 tumors were isolated from *Tie2-Cre^ERT2^*; *Wt1^Lox/Lox^* mice treated with Tamoxifen or vehicle. Briefly, lung and tumor tissues were cut into small fragments and digested with 1 mg/mL collagenase A and 100 IU/mL type I DNase (Roche Diagnostics, Meylan, France) for 45 min at 37 °C. ECs were then purified from the cell suspension using a rat anti-CD31 antibody (clone MEC 13.3; BD Biosciences, San Jose, CA, USA) conjugated to Dynabeads (Life Technologies, Courtaboeuf, France) using a magnetic particle concentrator and cultured on 0.2% type I collagen-coated plates (Sigma Aldrich, St. Louis, MO, USA) in DMEM medium supplemented with 20% FCS, 100 IU/mL penicillin, and 100 µg/mL streptomycin. Endothelial cell purity was confirmed by FACS analysis using Alexa Fluor 647 anti-mouse VE-cadherin antibody (Clone: BV13; BioLegend, San Diego, CA, USA) and anti-mouse Alexa Fluor 488 Fab′2 recognizing the VE-cadherin antibody.

### 2.4. RT-PCR and Quantitative RT-PCR

Total RNA was isolated using the Trizol reagent (Invitrogen). First-strand cDNA synthesis was performed with 0.5 µg of total RNA using the Thermo Scientific Maxima First Strand cDNA synthesis kit (Thermo Scientific, Illkirch, France). The reaction product was diluted to 100 µL and 1 µL of the diluted reaction product was taken for real time RT-PCR amplification (StepOne plus, Applied Biosystems, Foster City, CA, USA) using the SYBR^®^ Select Master Mix (Applied Biosystems). Expression of each gene was normalized to the respective arithmetic means of *Gapdh* (NM_001289726.1), *Actnb* (NM_007393.5), and *Rplp0* (NM_007475.5) expression. Vegf isoform expression was determined as described using identical PCR conditions and primers [18,26]. Vegf PCR products were analyzed on agarose gels with 100 bp molecular marker (Life Technologies) to verify that the PCR products correspond to the predicted size. Primer sequences are listed in Table 1.

### 2.5. Tissue Samples and Immunohistology

Paraffin-embedded samples, cut at 3 µm, were used for immunohistochemical detection of SRSF1. For immunohistology, after heat-mediated antigen retrieval at pH 6 and quenching of endogenous peroxidase activity, SRSF1 was detected using a polyclonal goat antibody from Santa Cruz (Heidelberg, Germany) in a dilution of 1:100, and the EnVisionTM Peroxidase/DAB Detection System from Dako (Trappes, France). For human melanoma tumors, the DAB substrate was replaced by VIP substrate (Vector, CliniSciences, Nanterre, France). The study adhered to the principles of the Declaration of Helsinki and to Title 45 of the U.S. Code of Federal Regulations (Part 46, Protection of Human Subjects). In total, 40 paraffin-embedded human tumor samples (10 lung cancers, 10 melanomas, 10 pancreas cancers, and 10 colon cancers) were used for this study. Negative controls were obtained by incubation of samples with a goat IgG Control (Invitrogen, Courtaboeuf, France). Sections were counterstained with Hematoxylin (Dako Trappes, France) and analyzed by two independent investigators, one of them was an experienced pathologist. Slides were viewed under an epifluorescence microscope (DMLB, Leica, Wetzlar, Germany) connected to a digital camera (Spot RT Slider, Diagnostic Instruments, Sterling Heights, MI, USA).

### 2.6. Cloning and Transient Transfection Experiments

The Srsf1 promoter construct was a kind gift of S.V. Graham [28]. The Srpk1 promoter construct was described previously [19]. As vector backbone, pGl3 basic (Promega, Charbonnières-les-Bains, France) was used for both constructs. A co-transfected beta-Galactosidase construct was used to normalize for differences in transfection efficiencies [29]. Each promoter construct was co-transfected with Wt1(−KTS) or Wt1(+KTS) expression constructs in C166 mouse endothelial cells using Lipofectamine LTX reagent (Thermo Scientific, Courtaboeuf, France) (*n* = 12 each). A putative Wt1 binding site was deleted from the Srsf1 promoter construct using the Quik Change II site directed mutagenesis kit (Stratagene, Agilent Technologies, Massy, France) with the following oligonucleotides: 5′-GTGGGGAGGGTGACGTTGAACGTAGCCCT-3′; antisense: reverse complement. The deletion construct for the Srpk1 promoter has been published recently [19]. Deletion constructs were again co-transfected with Wt1(−KTS) or Wt1(+KTS) expression constructs (*n* = 12 each).

### 2.7. Chromatin Immunoprecipitation Assay

Chromatin immunoprecipitation (ChIP) assay was performed on C166 cells using manufacturer instructions (Millipore, Burlington, MA, USA) as described [22,29]. One microgram of the following antibodies was used for each incubation of the DNA–protein complexes: WT1, rabbit monoclonal (ab52933, Abcam, Cambridge, UK), WT1 rabbit polyclonal (sc-192, Santa Cruz Biotechnology, Dallas, TX, USA), Acetyl-and Histone H3 (06-599, Upstate, Millipore, Burlington, MA, USA). Normal rabbit serum served as a negative control and dilutions of the input sample as positive control. The following primers were used: Srsf1 promoter, 5′-TACCAAACGGCTGGTCACTC-3′ (forward), 5′-ACAGCGATTCGATCCCAACA-3′ (reverse); Srsf1 3′UTR, 5′-TGGGCTAAAGTTGAATTGCAT -3′ (forward), 5′-ACCACAAACACCCCCAACAT-3′ (reverse). PCR products were electrophoresed on 4% agarose gels. Alternatively, samples were used in quantitative PCRs (*n* = 3 each). Fold enrichment was calculated from CT values relative to the input signal of each experiment set to 100% [24].

### 2.8. Statistical Analysis

Data are expressed as means ± S.E.M. Student’s *t*-tests (Instat, GraphPad, San Diego, CA, USA) were performed to determine statistical significance. A *p*-value of less than 0.05 was considered significant.

## 3. Results and Discussion

### 3.1. Wt1, Srpk1, and Srsf1 Are highly Expressed in Tumor versus Lung Endothelium

**Figure 1:**
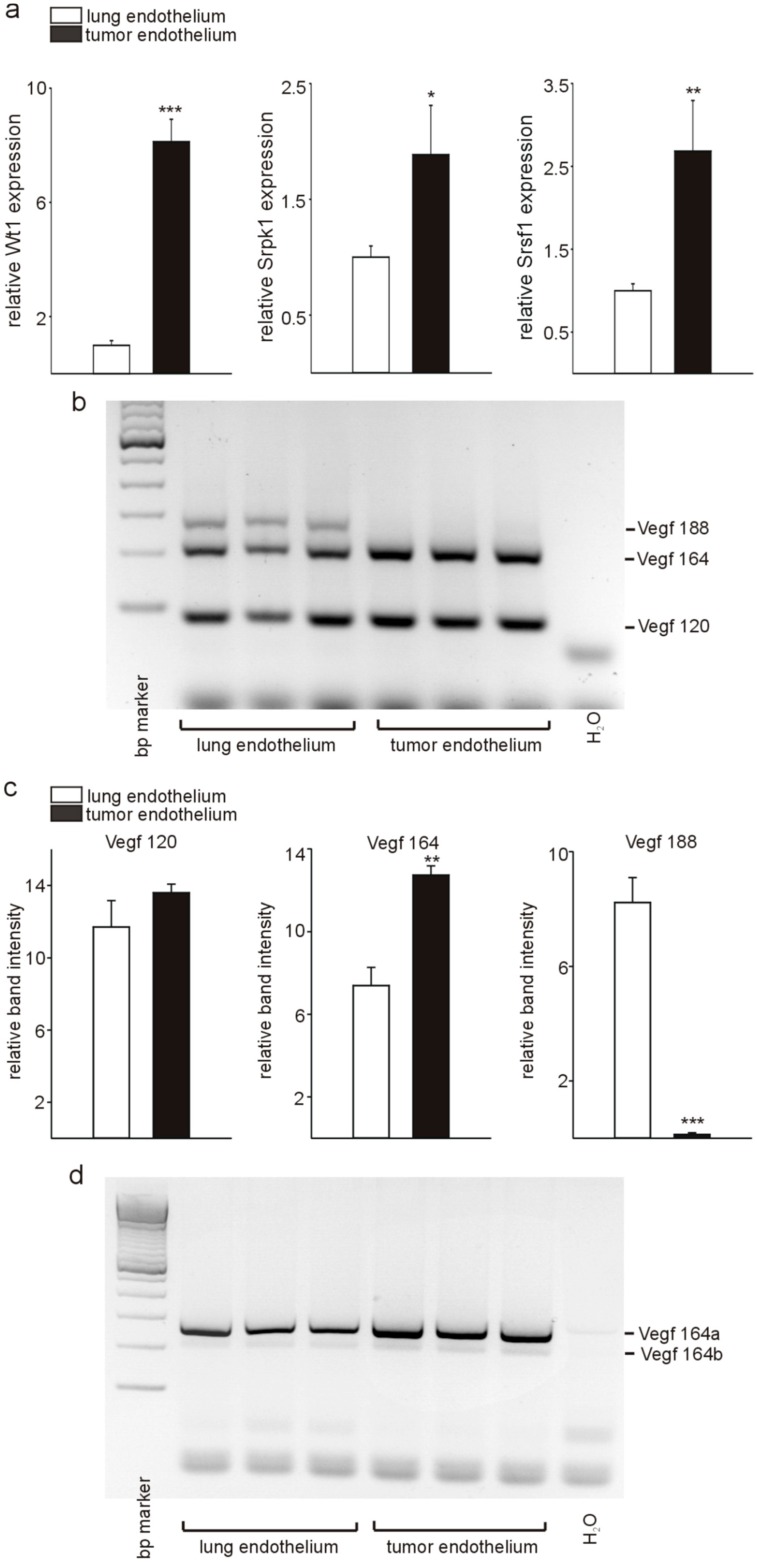
Differential expression of Wt1, Srpk1, and Srsf1, and Vegf splicing in tumor versus normal lung endothelium. (**a**) Quantitative RT-PCR analysis of Wt1, Srpk1, and Srsf1 expression in isolated tumor versus lung endothelial cells (*n* = 3 each). (**b**) Detection of Vegf isoforms (*n* = 3) was performed by RT-PCR followed by agarose gel electrophoresis as described [18]. A representative example is shown. The different molecular weight isoforms are indicated (Vegf 188: 293 bp; Vegf 164: 221 bp; Vegf 120: 89 bp). (**c**) Quantification of the different Vegf isoforms in isolated tumor and lung endothelial cells. (**d**) Detection of the Vegf 164a (281 bp) and Vegf 164b (215 bp) isoforms was performed by RT-PCR followed by agarose gel electrophoresis as reported previously [26] (*n* = 3). Data are expressed as means ± S.E.M. * *p* < 0.05, ** *p* < 0.01, *** *p* < 0.001.

We isolated endothelial cells from lungs and tumors of wild-type mice by CD31 labeling and magnetic cell sorting and analyzed Wt1, Srpk1, and Srsf1 expression by quantitative RT-PCR (Figure 1a). In agreement with our previous reports [22,29], we found quantitative significantly higher Wt1 expression in endothelial cells from tumors compared to normal lung endothelium. Although WT1 has been reported to suppress SRPK1 expression in human podocyte cell lines [19], we found that Srpk1 expression in tumor endothelium was higher compared to lung endothelial cells and also that Srsf1 expression was increased in tumor endothelial cells. Higher Srsf1 mRNA expression was equally unexpected as the established function of Srpk1 is the phosphorylation of Srsf1 [30], which results in nuclear import of Srsf1 [31] and affects VEGF splicing [19]. Therefore, we next analyzed VEGF splice variants, i.e., Vegf 188, Vegf 164, and Vegf 120 as well as Vegf 164 a and Vegf 164 b by reverse transcription (RT)-PCR using established oligonucleotides and PCR conditions [18,26] (Figure 1b–d). The Vegf 188 variant was highly expressed in lung endothelium, but not in tumor endothelial cells, while the angiogenic Vegf 164 variant showed increased expression in tumor compared to normal lung endothelial cells. No significant differences were observed for Vegf 120 between the two cell types (Figure 1b, and quantification in Figure 1c). The antiangiogenic Vegf 164b isoform was barely detectable in lung endothelial cells, and the fraction compared to total Vegf 164 was also not different in tumor endothelium (Figure 1d). The finding that Vegf 188 is only expressed in the lung is in agreement with the literature [32,33]. Bacic et al. reported predominant expression of Vegf 188 in rat heart and lung, while. in all other tissues, this isoform was the least abundant. Unfortunately, only normal tissues, but no tumors have been investigated in this study [32]. In the lung, VEGF is expressed on alveolar epithelial type II cells, vascular endothelium and alveolar macrophages, but isoform expression in the different cell types has not been reported [34]. The role of VEGF 189 for cancer progression is highly controversial with reports showing the highest vessel density and poorest prognosis in tumors overexpressing VEGF 189 [35,36], while others showed less metastases and improved survival in breast cancer cell lines overexpressing VEGF 189 [37]. High Vegf 164 expression in tumor endothelium is in line with a pro-angiogenic phenotype of endothelial cells in tumors.

In agreement with the high Srpk1 and Srsf1 expression in tumor endothelial cells, we detected increased levels of Vegf 164a [19,38,39], but only low expression of Vegf 164 b in tumor as well as lung endothelial cells. It has been described that, in the normal kidney, colon, bladder smooth muscle, lung, and pancreatic islets, VEGF b isoforms predominate, while, in colorectal carcinoma, bladder cancer, melanoma, prostate cancer cell lines, and de-differentiated podocytes, angiogenic VEGF a isoforms predominate [19,40,41] (reviewed in [1]). To our knowledge, little is known about the expression of Vegf a and Vegf b isoforms in endothelial cells of different vascular beds. As pro-angiogenic Vegf is required for endothelial cell survival [42], it is not surprising that we mainly detected angiogenic Vegf a variants in endothelial cells. Unfortunately, given the relatively small number of cells isolated using our magnetic separation protocol, we were not able to confirm Vegf 164 a and Vegf 164 b expression differences between tumor and lung endothelial cells on the protein level by ELISA.

### 3.2. Srsf1 Protein Is Differentially Expressed in Normal Tissue Endothelium Compared to Tumor Endothelium

**Figure 2:**
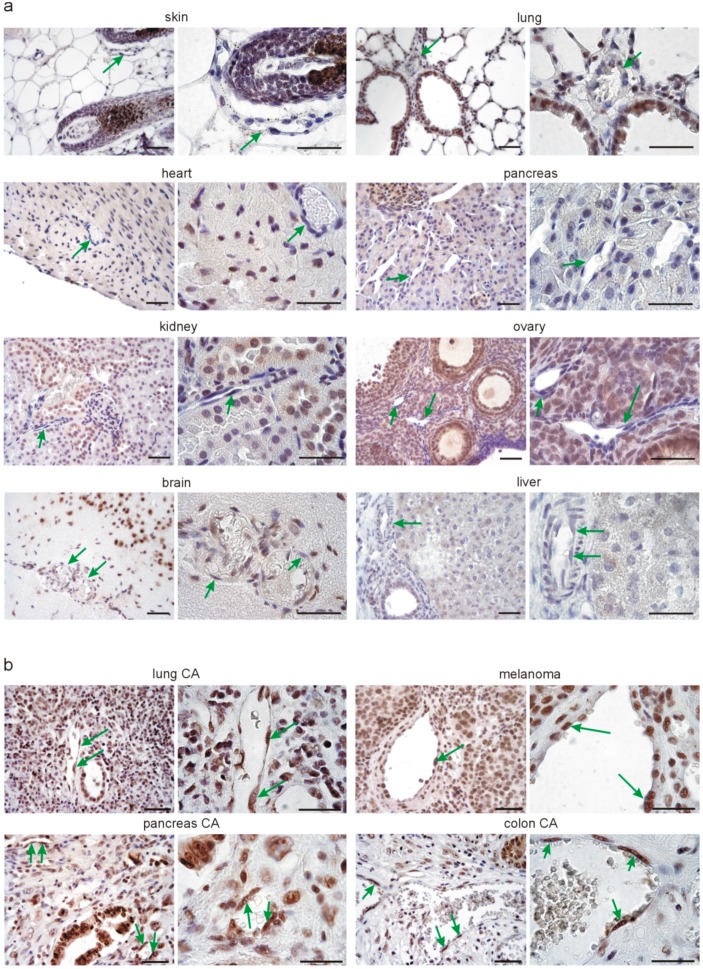
Srsf1 expression in different healthy adult mouse tissues and different human tumor types. (**a**) Representative examples of Srsf1 immunostaining (DAB substrate, brown) in skin, lung, heart, pancreas, kidney, ovary, brain, and liver sections of adult mice (*n* = 3 each). Nuclei were counterstained with hematoxylin (blue). For each organ, one example and a higher magnification are shown. Note the nuclear localization of Srsf1 in different cell types of the organs. Green arrows point to vascular endothelial cells in the different organs, which are rarely Srsf1-positive. (**b**) Representative examples of SRSF1 expression in human lung cancer, melanoma, pancreas, and colon cancer sections (*n* = 10 each). Immunostaining for SRSF1 was visualized with DAB substrate (brown) and the nuclei counterstained with hematoxylin (blue). Note in human tumors the high nuclear SRSF1 expression in tumor and endothelial cells. Green arrows point to endothelial cells. Scale bars indicate 50 µm.

Since we detected lower Srpk1 and Srsf1 expression in isolated endothelial cells from lungs compared to tumors, we next addressed the questions whether this corresponds to the situation in vivo and might represent a more general phenomenon in normal healthy tissues compared to tumor samples. We used immunohistochemistry for Srsf1 on multiple normal mouse tissue samples (Figure 2a) and on human lung, pancreas, and colon cancer and melanoma sections (Figure 2b). In normal tissues, we detected strong nuclear immunoreactivity for Srsf1 in hair bulbs of the skin, the alveolar epithelium of the lung, cardiomyocytes and fibroblasts in the heart, mainly endocrine, but also exocrine cells of the pancreas, tubules and glomeruli of the kidney, follicles and stroma of the ovary, and some neurons in the brain. Hepatocytes of the liver showed weaker nuclear staining for Srsf1. In all these analyzed normal tissues, Srsf1 was rarely detectable in the nuclei of endothelial cells (green arrows in Figure 2a). To our knowledge, Srsf1 expression in such a variety of different normal mouse tissues has not been reported before. In contrast, in different tumors, i.e., lung, pancreas, and colon cancer, and in melanomas, intense nuclear SRSF1 staining was observed in tumor cells, which is in agreement with the reported overexpression of SRSF1 in different cancer types and its function as proto-oncogene [43,44]. In agreement with our results on isolated endothelial cells, we detected nuclear SRSF1 expression in most of the endothelial cells in tumors of different origin (arrows in Figure 2b).

### 3.3. Inducible Vascular-Specific Knockout of Wt1 Abolishes Nuclear Endothelial Srsf1 Expression

**Figure 3:**
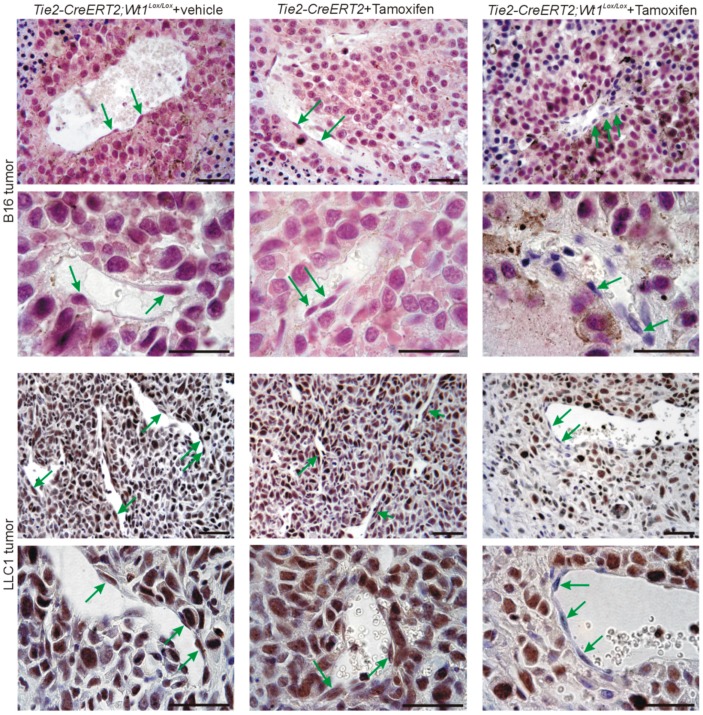
Srsf1 expression in different mouse tumor types. B16 melanoma tumors (**top**) were produced by subcutaneous injection of tumor cells in *Tie2-CreERT2*; *Wt1^Lox/Lox^* mice treated with vehicle (*n* = 13) or *Tie2-CreERT2* animals (*n* = 11) treated with Tamoxifen as controls and in *Tie2-CreERT2*; *Wt1^Lox/Lox^* mice treated with Tamoxifen to knockout *Wt1* (*n* = 20). LLC1 lung cancer cell tumors (**bottom**) were produced by subcutaneous injection of tumor cells in *Tie2-CreERT2*; *Wt1^Lox/Lox^* mice treated with vehicle (*n* = 9) or *Tie2-CreERT2* animals (*n* = 8) treated with Tamoxifen as controls and in *Tie2-CreERT2*; *Wt1^Lox/Lox^* mice treated with Tamoxifen to knockout *Wt1* (*n* = 11). Srsf1 immunostaining was visualized with VIP (purple) for melanoma tumors and DAB (brown) for LLC1 tumors. Nuclei were counterstained with hematoxylin (blue). Note the high expression and nuclear localization of Srsf1 in tumor and endothelial cells in *Tie2-CreERT2*; *Wt1^Lox/Lox^* mice treated with vehicle and *Tie2-CreERT2* animals treated with Tamoxifen and the absence of Srsf1 in endothelial cells of *Tie2-CreERT2*; *Wt1^Lox/Lox^* animals treated with Tamoxifen. Green arrows point to endothelial cells. Scale bars indicate 50 µm.

As it has been described that Wt1 regulates Srpk1 [19] and Srpk1 phosphorylates Srsf1, which results in nuclear import of the protein [30,31], we were interested in deciphering the role of Wt1 for the high nuclear Srsf1 expression in tumor endothelial cells. For this purpose, we used Tie2-CreERT2 mice crossed with Wt1^Lox/Lox^ animals as reported before [22], induced vessel-specific knockout of Wt1 by Tamoxifen injection, and implanted syngenic B16 melanoma or LLC1 lung cancer cells subcutaneously. Tie2-CreERT2; Wt1^Lox/Lox^ animals injected with vehicle (sunflower oil) and Tie2-CreERT2 transgenic animals injected with Tamoxifen before tumor cell implantation served as controls. Tumor samples were collected before complete regression occurs in this model [22] and analyzed for Srsf1 expression by immunohistochemistry. Comparable to human tumors, significant nuclear Srsf1 expression was detected in tumor and endothelial cells in control animals (Figure 3, left and middle), but Srsf1 was barely detectable in endothelial cells of tumors from Tie2-CreERT2; Wt1^Lox/Lox^ animals injected with Tamoxifen (green arrows in Figure 3, right). Expression of Srsf1 in B16 melanoma cells and LLC1 lung tumor cells was unaffected by vessel-specific knockout of Wt1. The overall aspect of reduced immunoreactivity for Srsf1 in tumors of Tie2-CreERT2; Wt1^Lox/Lox^ + Tamoxifen animals compared to controls might be attributed to increased lymphocyte infiltration, necrosis, and larger fibrotic areas with reduced number of tumor cells as reported recently [22].

### 3.4. Knockout of Wt1 in Tumor Endothelium Affects Srpk1, and Srsf1 Expression and Vegf Splicing

To determine quantitative expression differences for Wt1, Srpk1, and Srsf1 and to estimate differences in Vegf splicing, we used a comparable approach as mentioned above and isolated endothelial cells by magnetic sorting from tumors of *Tie2-CreERT2*; *Wt1^Lox/Lox^* animals treated with Tamoxifen and vehicle-injected controls. Quantitative RT-PCR analyses revealed significantly reduced Wt1 expression in endothelial cells from tumors of *Tie2-CreERT2*; *Wt1^Lox/Lox^* mice injected with Tamoxifen versus vehicle-treated controls to a comparable extend as reported earlier [22].

**Figure 4:**
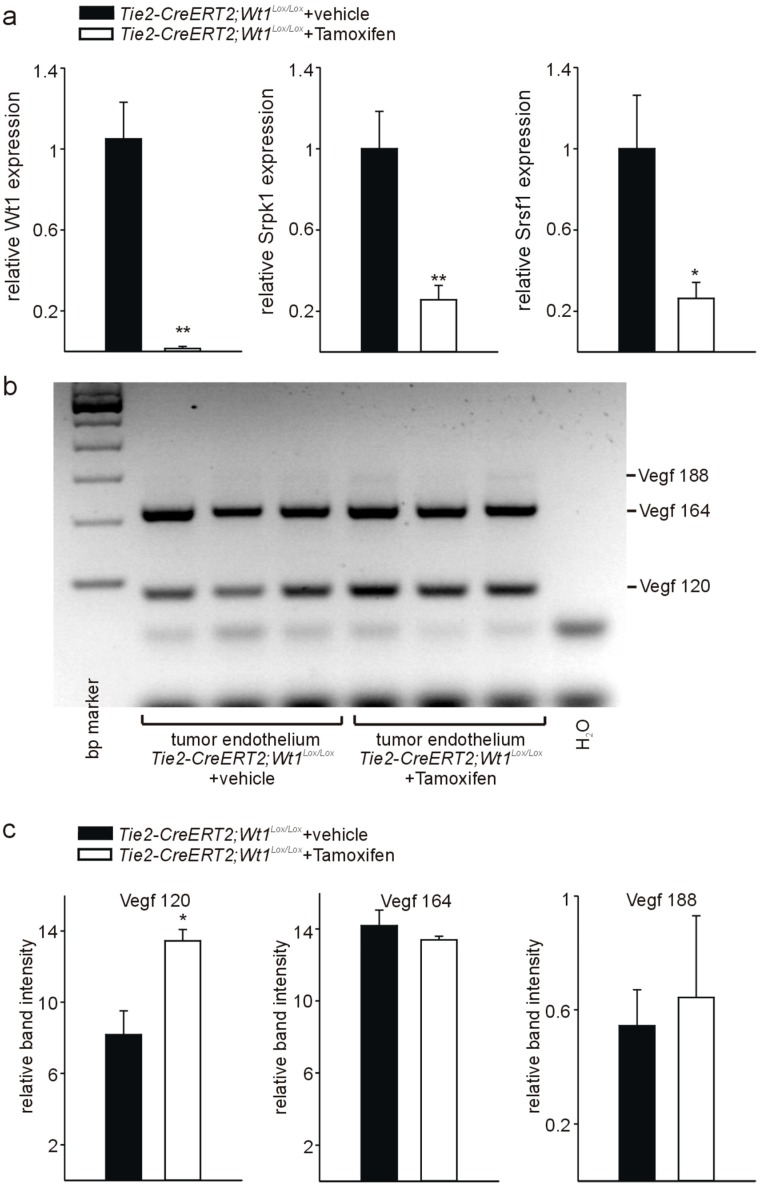
Reduced expression of Wt1, Srpk1, and Srsf1 and altered Vegf splicing in tumor endothelial cells of *Tie2-Cre^ERT2^*; *Wt1^Lox/Lox^* mice treated with Tamoxifen compared to vehicle-treated controls. (**a**) Expression levels of Wt1, Srpk1, and Srsf1 were determined by quantitative RT-PCR on isolated endothelial cells of tumors from *Tie2-Cre^ERT2^*; *Wt1^Lox/Lox^* mice treated with Tamoxifen compared to vehicle-treated controls (*n* = 6 each). Expression of each gene was normalized to the respective arithmetic means of *Gapdh*, *Actnb,* and *Rplp0* expression. (**b**) Expression of the different Vegf isoforms (Vegf 188: 293 bp; Vegf 164: 221 bp; Vegf 120: 89 bp) was determined on isolated endothelial cells (*n* = 3 each) as described [18]. (**c**) Quantification of relative band intensities revealed relative higher Vegf120 isoform levels, while Vegf164 and Vegf188 were not significantly affected by the knockout of Wt1. Data are expressed as means ± S.E.M. * *p* < 0.05, ** *p* < 0.01.

In addition, Srpk1 and Srsf1 RNA expression was lower in endothelial cells from tumors of *Tie2-CreERT2*; *Wt1^Lox/Lox^* mice injected with Tamoxifen compared to controls (Figure 4a). Next, we determined Vegf isoform expression in endothelial cells from tumors of *Tie2-CreERT2*; *Wt1^Lox/Lox^* mice injected with Tamoxifen compared to vehicle-treated controls by PCR as described [18]. The Vegf 188 isoform was barely detectable, while the Vegf 164 and Vegf 120 represented the major isoforms expressed in endothelial cells (Figure 4b,c). This is in agreement with the results presented in Figure 1. Knockout of Wt1 in endothelial cells in the tumors did not significantly affect the Vegf 188 and Vegf 164 isoforms, but induced a relative increase in the Vegf 120 isoform (Figure 4c). No significant differences were observed for the Vegf 164a and Vegf 164b isoforms (data not shown).

**Figure 5:**
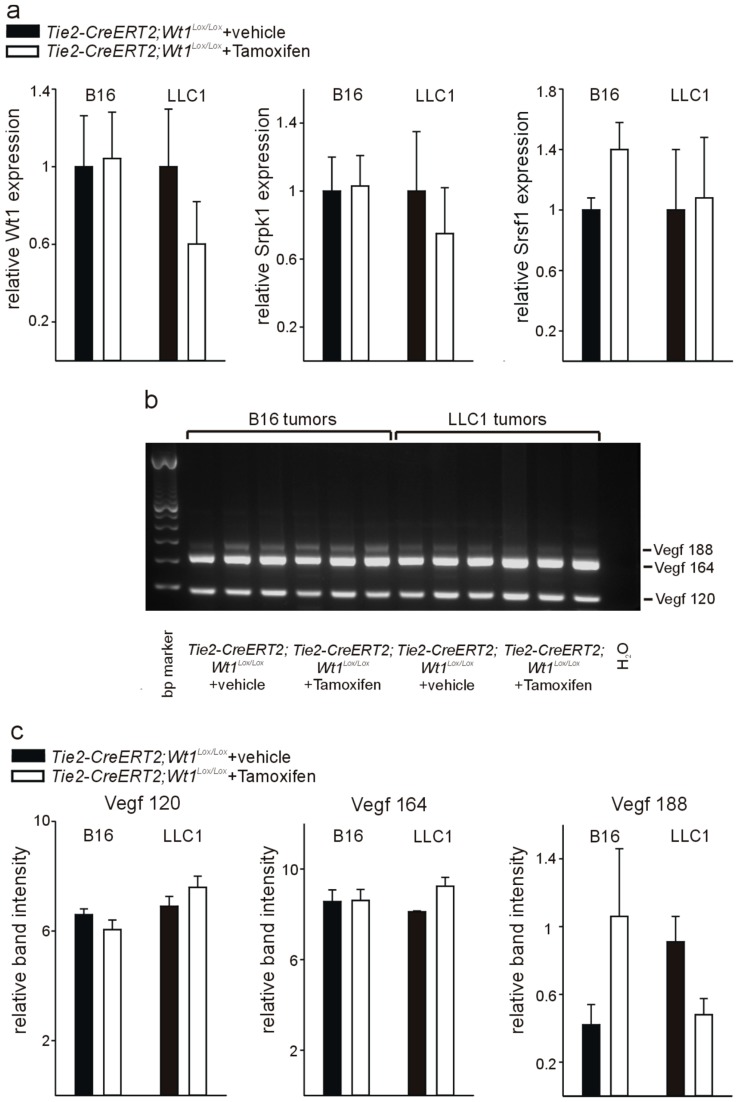
Determination of Wt1, Srpk1 and Srsf1 expression and Vegf isoform distribution in whole tumor samples from *Tie2-Cre^ERT2^*; *Wt1^Lox/Lox^* mice treated with Tamoxifen compared to vehicle-treated controls. (**a**) Expression levels of Wt1, Srpk1 and Srsf1 were determined by quantitative RT-PCR on B16 and LLC1 tumor samples from *Tie2-Cre^ERT2^*; *Wt1^Lox/Lox^* mice treated with Tamoxifen compared to vehicle-treated controls (*n* = 6 each). Expression of each gene was normalized to the respective arithmetic means of *Gapdh*, *Actnb,* and *Rplp0* expression. (**b**) Vegf isoforms (Vegf 188: 293 bp; Vegf 164: 221 bp; Vegf 120: 89 bp) were determined by PCR as described [18] in samples of B16 and LLC1 tumors isolated from *Tie2-Cre^ERT2^*; *Wt1^Lox/Lox^* mice treated with Tamoxifen compared to vehicle-treated controls (*n* = 3 each). (**c**) Quantification of relative band intensities for the different Vegf isoforms.

To investigate whether these observed differences are specific for endothelial cells or reflect differences in the tumors of *Tie2-CreERT2*; *Wt1^Lox/Lox^* mice injected with Tamoxifen compared to vehicle-treated controls, we isolated RNA from B16 melanoma and LLC1 tumors from the two groups of mice. For both tumor types, no significant expression differences for Wt1, Srpk1, and Srsf1 could be detected by quantitative RT-PCR from whole tumor RNA of *Tie2-CreERT2*; *Wt1^Lox/Lox^* + Tamoxifen animals compared to controls (Figure 5a). In addition, no significant differences in Vegf 188, Vegf 164, and Vegf 120 isoform expression could be detected in B16 and LLC1 tumors of *Tie2-CreERT2; Wt1^Lox/Lox^* + Tamoxifen animals and vehicle-treated controls (Figure 5b,c). As endothelial cells represent only approximately 6% of the cells in the investigated tumor types [22], it is not surprising that, although we observed significant differences for Wt1, Srpk1, Srsf1, and Vegf isoforms in isolated endothelial cells, this is not reflected in whole tumor samples. Furthermore, it supports the specificity of the findings for endothelial cells.

Increased relative expression of the Vegf 120 isoform has already been reported in Wt1-deficient hematopoietic progenitor cells [18]. As hematopoietic progenitor cells contribute to endothelial cells [45], our result of increased relative expression of the Vegf 120 isoform in endothelial cells with knockout of Wt1 is in agreement with this study. Interestingly, the increase in Vegf 120 in Wt1-deficient hematopoietic progenitor cells has been linked to apoptosis and reduced hematopoietic potential of these cells, which could be rescued by Vegf treatment [18]. We reported increased apoptosis also in endothelial cells with knockout of Wt1 [22]. Thus, our results in endothelial cells correspond also in this aspect to hematopoietic progenitor cells. As Vegf isoforms expression showed no significant differences in whole tumor RNA preparations, but increased Vegf 120 was detectable in isolated endothelial cells, our results support the hypothesis that mainly endogenous VEGF in endothelial cells is important to maintain endothelial cell homeostasis [21].

Relatively moderate changes in Vegf isoform distribution upon knockout of Wt1 and clear reduction in Srpk1 and Srsf1 expression might be explained by a multitude of splicing factors, which beside Srsf1 act in endothelial cells [46]. Nevertheless, as it has been shown that normal VEGF levels and isoform expression are required for normal embryonic development [7,8,9,10,11], it is not surprising that changes in Vegf isoform distribution upon knockout of Wt1 in endothelial cells contribute to endothelial cell apoptosis and vascular regression in our tumor models [22]. In addition, we cannot rule out the possibility that Vegf isoform protein level differences were more pronounced, which we could not determine due to the limited amount of material.

### 3.5. Wt1 Activates Srpk1 and Srsf1 in Endothelial Cells

**Figure 6:**
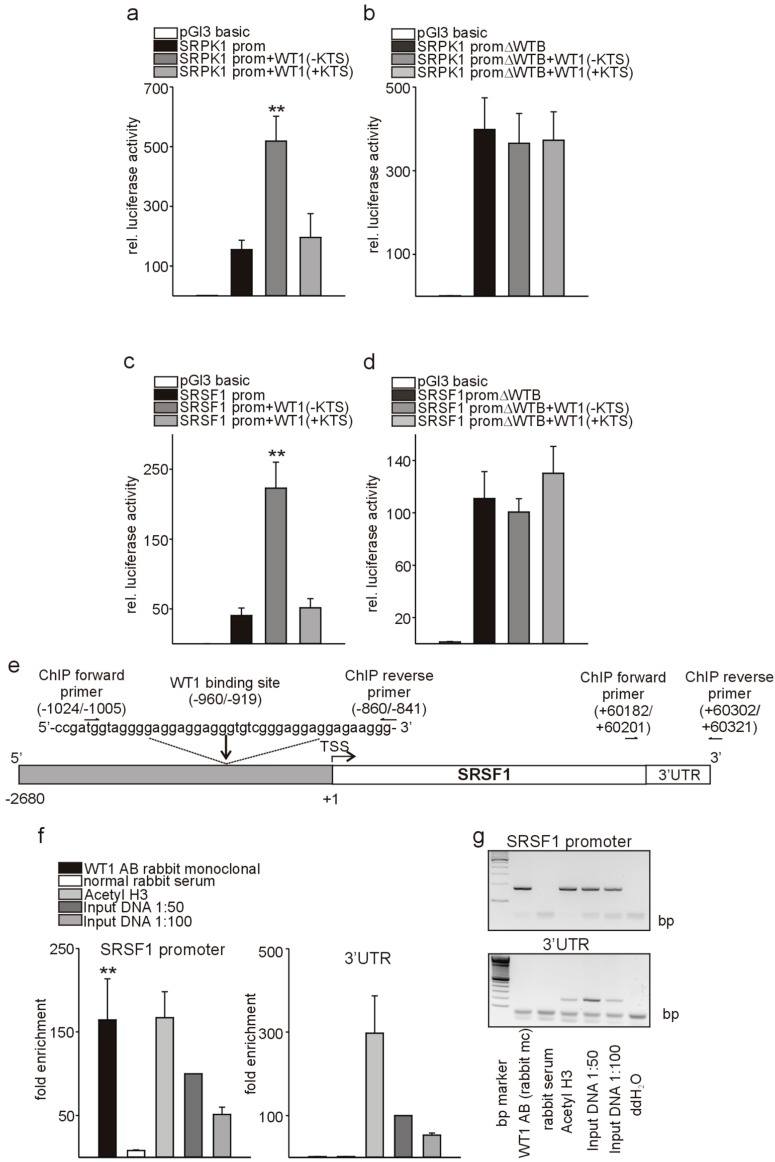
WT1 activates SRPK1 and SRSF1 in endothelial cells. (**a**) Relative luciferase activity of a reporter construct carrying the SRPK1 promoter [19] in the presence of WT1(−KTS) or WT1(+KTS) expression constructs. Transient transfections were performed using C166 cells (*n* = 12 each). The promoterless luciferase expression construct (pGl3basic) served as a negative control. Co-transfected beta-Galactosidase was used to normalize for differences in transfection efficiencies. (**b**) Relative luciferase activity of a SRPK1 promoter reporter construct with deletion of the identified WT1-binding site (ΔWTB) in the presence of WT1(−KTS) or WT1(+KTS) expression constructs (*n* = 12 each). (**c**) The published SRSF1 promoter construct [28] in pGl3basic was co-transfected with WT1(−KTS) and WT1(+KTS) expression constructs in C166 endothelial cells (*n* = 12). Beta-Galactosidase served to normalize for differences in transfection efficiency. (**d**) ΔWTB indicates reporter constructs with deletion of the predicted WT1-binding site in the SRSF1 promoter construct. Transfection experiments were performed as in (c) (*n* = 12). (**e**) Schematic representation of the putative WT1-binding site in the SRSF1 promoter. Positions of the cloned promoter relative to the transcription start site, the position and sequence of the putative WT1-binding site and positions of the oligonucleotides used for CHIP analyses are indicated. For the promoter-deletion construct (d), the indicated WT1-binding site was removed from the promoter reporter construct. (**f**) Chromatin immunoprecipitation (ChIP, *n* = 4) was performed using a rabbit monoclonal antibody against WT1 or anti-acetylhistone H3 antibody as positive control. Normal rabbit serum served as a negative control. Input DNA was used as additional positive control for quantitative PCRs on the SRSF1 promoter and the respective 3′UTR sequence. (**g**) Representative agarose gel photographs of semi-quantitative ChIP PCR experiments for the SRSF1 promoter sequence (upper picture) and the respective (3′UTR). Data are expressed as means ± S.E.M. ** indicates *p* < 0.01.

As WT1 represses SRPK1 in podocytes [19], but we observed increases in Srpk1 and Srsf1 in tumor endothelial cells with high Wt1 expression compared to lung endothelial cells and a decrease of Srpk1 and Srsf1 expression upon endothelial-specific knockout of Wt1, we investigated whether Wt1 might be an activator instead of a repressor of Srkp1 and Srsf1 promoter activity in endothelial cells. For this purpose, we transiently co-transfected Srpk1 or Srsf1 promoter constructs [19,28] in the pGl3 basic luciferase reporter vector together with WT1(−KTS) or WT1(+KTS) expression constructs in C166 endothelial cells. These two WT1 variants differ by the presence/absence of three amino acids (KTS) in the zinc finger domain of the molecule. SRPK1 and SRSF1 promoter activity was stimulated by WT1(−KTS). In contrast, WT1(+KTS), which has a role in posttranscriptional RNA processing and pre-mRNA splicing rather than transcriptional regulation [47,48], did not significantly change promoter activities (Figure 6a–d). A SRPK1 promoter construct with deletion of the identified WT1-binding site [19] showed higher basal activity compared to the wild-type promoter construct when transfected in C166 endothelial cells. This might indicate that besides WT1 other transcription factors, which act as repressors bind to this region. Co-transfection of the SRPK1 promoter construct with deletion of the identified binding site [19] with WT1(−KTS) or WT1(+KTS) expression constructs in C166 endothelial cells abolished activation of the promoter construct (Figure 6b). Interestingly, the SRSF1 promoter contained multiple repetitions of known WT1-binding elements (ggagg) (Figure 6e). Deletion of this predicted WT1-binding site in the SRSF1 promoter construct resulted in higher basal activity, but abolished activation by WT1(−KTS) (Figure 6d). As physical interaction of WT1 with the SRPK1 promoter has been shown already [19], we focused for chromatin immunoprecipitation assays (CHIP) only on the SRSF1 locus. We used a rabbit monoclonal antibody against WT1, which confirmed direct binding of WT1 to the SRSF1 promoter, but not to 3′ UTR sequence of SRSF1. An antibody against Acetyl-histone 3 and input DNA served as positive controls and normal rabbit serum as negative control (Figure 6e–g).

Inhibition of SRPK by WT1 in podocytes, but activation in endothelial cells, is not surprising as it is well known that WT1 might act as activator or repressor of transcription in different cell types (reviewed in [49,50]). Co-factors that might be involved in this differential regulation in podocytes and endothelial cells remain to be identified.

## 4. Conclusions

We show here that Wt1, Srpk1, Srsf1, and the angiogenic Vegf 164a isoform are highly expressed in tumor endothelial cells compared to normal lung endothelium. Knockout of Wt1 in endothelial cells reduces Srpk1 and Srsf1 expression and induces a shift towards the Vegf 120 isoform. Wt1 acts as an activator instead of as a repressor of Srpk1 in endothelial cells. Wt1 double secures VEGF splicing as it directly activates Srpk1 and Srsf1. Inhibition of Srpk1 and Srsf1, and alterations in Vegf splicing, which induce tumor endothelial cell apoptosis, might contribute to the antitumor activity upon targeting Wt1.

## Figures and Tables

**Table 1:** Primer Sequences.

Name	Sequence
Wt1 forward	CCA GCT CAG TGA AAT GGA CA
Wt1 reverse	CTG TAC TGG GCA CCA CAG AG
Vegfa Exon 4 forward	CAC AGC AGA TGT GAA TGC AG [18]
Vegfa Exon 8 reverse	CCT TCC TGC AGC CTG GCT C [18]
Vegf164a/b forward	CAG AAA ATC ACT GTG AGC CTT GTT [26]
Vegf164a/b reverse	ATT AAG GAC TGT TCT GTC AA [26]
Srpk1 forward	CCA AGT GAA GAT CGC AGA CC
Srpk1 reverse	TCT TCA GTG AAA TGC TTG TGC
Srsf1 forward	TCC GAG AAC AGA GTG GTT GTC
Srsf1 reverse	CAT ACA TCA CCT GCC TCA CG
Rplp0 forward	CAC TGG TCT AGG ACC CGA GAA G [27]
Rplp0 reverse	GGT GCC TCT GGA GAT TTT CG [27]
Gapdh forward	CCA ATG TGT CCG TCG TGG ATC T [27]
Gapdh reverse	GTT GAA GTC GCA GGA GAC AAC C [27]
Actb forward	CTT CCT CCC TGG AGA AGA GC [27]
Actb reverse	ATG CCA CAG GAT TCC ATA CC [27]
